# Synthon Substitution via C–I···π and C–I···N Halogen Bonds in Cocrystals of Anthracene-Based Organic Semiconductor Isosteres

**DOI:** 10.1021/acs.cgd.5c01513

**Published:** 2026-01-13

**Authors:** Ivan Bondarenko, Shivani Ahuja, Brian O. Patrick, Gonzalo Campillo-Alvarado

**Affiliations:** † Department of Chemistry, 6686 Reed College, Portland, Oregon 97202-8199, United States; ‡ Department of Chemistry, 8166 University of British Columbia, 2036 Main Mall, Vancouver, British Columbia V6T 1Z1, Canada

## Abstract

Cocrystallization is a versatile supramolecular synthetic strategy for tuning the properties of organic semiconductors (OSCs) and related polycyclic aromatic hydrocarbons (PAHs) by controlling their packing and architectures with suitable coformers. In this study, we demonstrate a supramolecular synthon substitution approach to afford cocrystals of 9,10-diphenylanthracene (**DPA**) and its isostere 9,10-dipyridylanthracene (**DPyA**) with halogenated coformers 1,2-diiodotetrafluorobenzene (**1,2-C**_6_ **I**_2_ **F**_4_), 1,4-diiodotetrafluorobenzene (**1,4-C**_6_ **I**_2_ **F**_4_), and 1,3,5-triiodotrifluorobenzene (**1,3,5-C**_6_ **I**_3_ **F**_3_). The strategy enables reliable replacement of [C–I···π] interactions in **DPA** cocrystals with [C–I···N] interactions in the corresponding **DPyA** cocrystals. Although coformers and substitutions alter the supramolecular architectures, the photophysical properties and molecular conformations of the OSC building blocks remain largely preserved. The results highlight synthon substitution as a reliable supramolecular design element that accelerates the derivatization of established OSCs and their isosteres, offering opportunities for property modulation.

## Graphical abstract

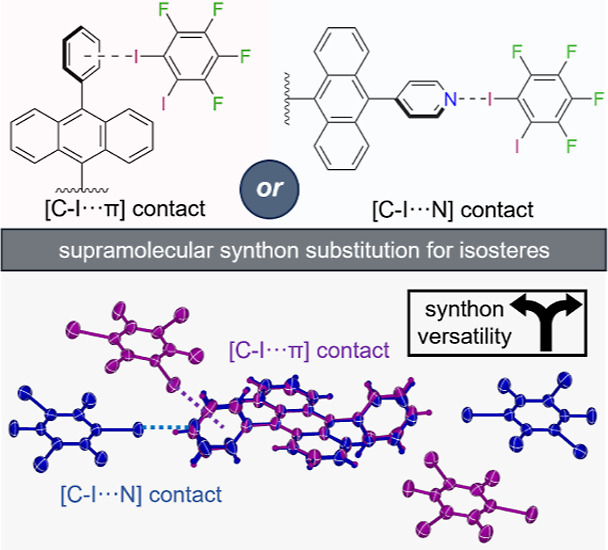

## 1 Introduction

The development of next-generation organic field-effect transistors (OFETs), solar cells, and sensors based on organic semiconductors (OSCs) requires precise control of molecular self-assembly and crystallization.^1–5^ Among OSCs, anthracenes and related polycyclic aromatic hydrocarbons (PAHs) are widely employed due to their extended π-conjugation and strong intermolecular interactions (e.g., π-stacking), which confer unique optical and electronic properties and generally result in high charge-carried mobilities.^6–9^ Despite their promise, the development of new OSCs often relies on complex, energy-intensive, and costly synthetic protocols.^10^

An alternative approach involves using weak, noncovalent interactions between OSCs and molecular coformers to direct the crystal packing of PAHs (i.e., cocrystallization). The supramolecular strategy has accelerated the diversification of architectures and properties of established OSCs.^11–19^ For example, halogenated coformers have been used to modulate aggregation of 9,10-diphenylanthracene (**DPA**),^20^ a chromophore widely employed in OFETs and light-emitting diode (LEDs) owing to its remarkable optical (e.g., highly fluorescent) and electronic properties.^21–23^ In the study, coformers organized **DPA** molecules through [C–*X*···π] contacts (*X* = Br, I), with pendant phenyl rings in **DPA**, resulting in cocrystals with tunable photoluminescence and electrochemiluminescence properties.^20^

Inspired by this work, we sought to explore whether a supramolecular synthon substitution strategy could be used to modulate the crystal packing and photophysical properties (e.g., fluorescence)^24^ of 9,10-dipyridylanthracene (**DPyA**), an isostere of **DPA** in which pyridyl rings replace the phenyl substituents on the anthracene core (Scheme 1), using a series of halobenzene coformers. Synthon substitution is a strategy in supramolecular synthesis^25–27^ that has recently been applied to generate nucleic acid base pairs by replacing hydrogen bonds with halogen bonds.^28^ Although both [C–I···π] and [C–I···N] interactions are individually well-documented in crystal engineering,^29–32^ the purposeful exploitation of their interchangeability as a predictive synthon substitution strategy, particularly across multiple coformer systems and structurally related organic semiconductor isosteres, remains largely unexplored. We hypothesize [C–I···π] contacts in **DPA** cocrystals could be reliably substituted by [C–I···N] halogen bonds in the **DPyA** analogues with a series of halobenzenes, enabling the development of multicomponent solids of OSC isosteres based on 9,10-diarylanthracenes. The development of cocrystals of **DPA** and **DPyA** could modulate photophysical properties (e.g., spectral intensity distribution, fluorescence quenching), as observed with cocrystals of 9,10-bis­((*E*)-2-(pyridin-4-yl)­vinyl)­anthracene.^12,33^ To the best of our knowledge, synthon substitution has not previously been deliberately employed as a design strategy for the cocrystal formation of anthracene-based OSC isosteres.

**Scheme 1:**
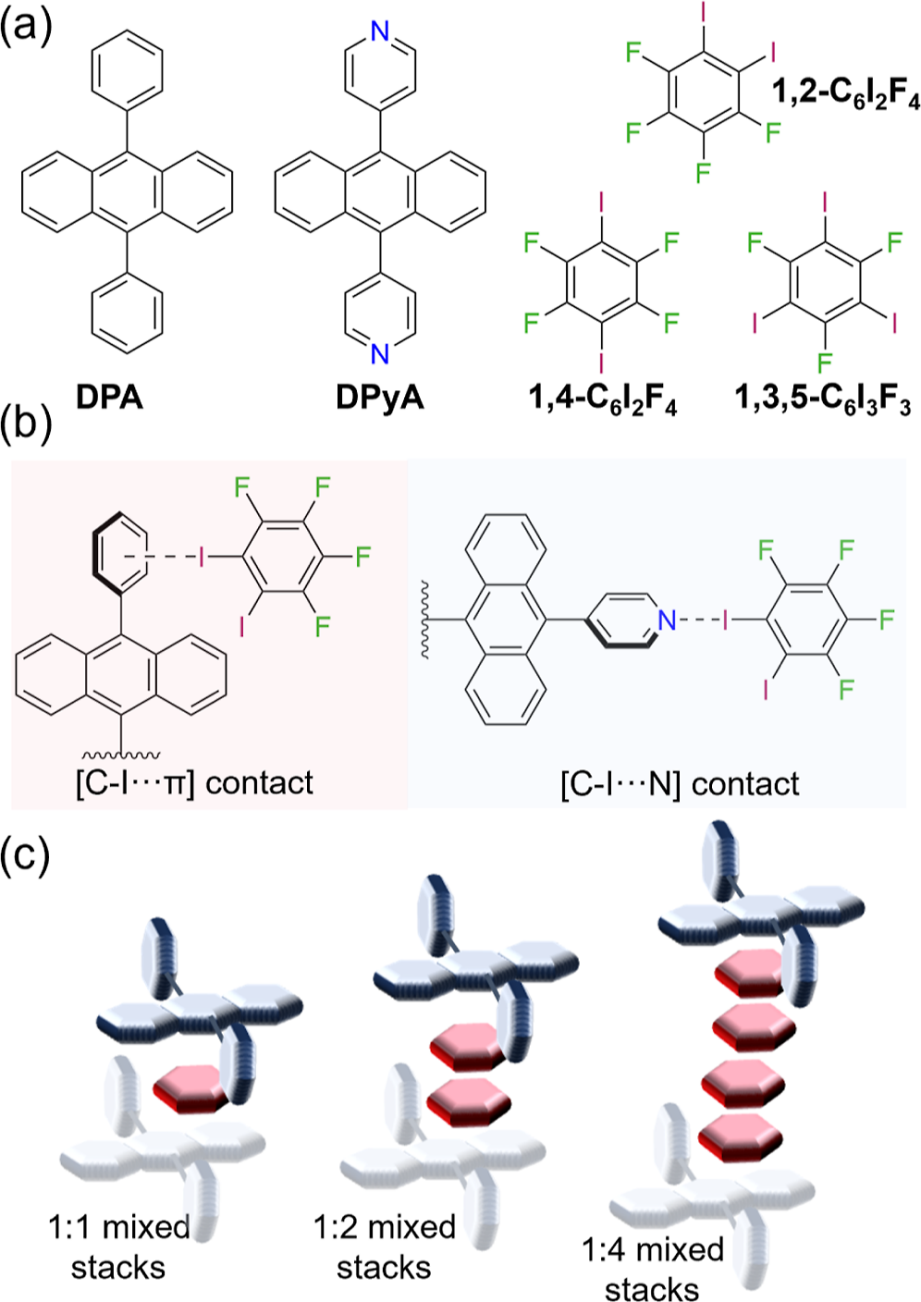
Supramolecular Synthon Substitution Strategy for Anthracene-Based Isosteres **DPA** and **DPyA** via Cocrystallization: (a) Molecular Building Blocks Used in This Study, (b) Supramolecular Synthons [C–I···π] and [C–I···N], and (c) Mixed-Stack Stoichiometries in Cocrystals

Here, we describe a solid-state ordering strategy for OSC isosteres using a supramolecular synthon substitution approach. Halobenzene coformers 1,2-diiodotetrafluorobenzene (**1,2-C**_6_ **I**_2_ **F**_4_), 1,4-diiodotetrafluorobenzene (**1,4-C**_6_ **I**_2_ **F**_4_), and 1,3,5-triiodotrifluorobenzene (**1,3,5-C**_6_ **I**_3_ **F**_3_) form cocrystals with **DPA** and **DPyA** via distinct halogen-bonding motifs (Scheme 1a). Cocrystals with **DPA** are primarily supported by [C–I···π] contacts with the pendant phenyl ring, whereas the **DPyA** isostere exhibits [C–I···N] halogen bonds (Scheme 1b). Notably, variations in coformer identity and synthon type result in mixed π-stacked assemblies with different stoichiometries (i.e., 1:1, 1:2, and 1:4 OSC/coformer ratios, Scheme 1c). While changes in coformers and synthons influence supramolecular architectures, the molecular conformations of **DPA** and **DPyA** molecules remain preserved across all of the solids, leading to comparable photophysical properties. The observations are supported by single-crystal and powder X-ray diffraction (SCXRD, PXRD) and Hirshfeld surface analyses and model energies.

## 2 Experimental Section

### 2.1 Cocrystal Synthesis

Chloroform, methanol, and acetonitrile were purchased from Sigma-Aldrich. Compounds **DPA**, **DPyA**, **1,3,5-C**_6_ **I**_3_ **F**_3_, **1,2-C**_6_ **I**_2_ **F**_4_, and **1,4-C**_6_ **I**_2_ **F**_4_ were purchased from AmBeed. All chemicals were used as received without further purification. Cocrystals **DPA·1,2-C**_6_ **I**_2_ **F**_4_, **DPA·1,3,5-C**_6_ **I**_3_ **F**_3_, **DPA·1,4-C**_6_ **I**_2_ **F**_4_, **DPyA·1,3,5-C**_6_ **I**_3_ **F**_3_, and **DPyA·1,2-C**_6_ **I**_2_ **F**_4_ were generated by heat- and sonication-assisted dissolution of the corresponding OSC isostere (0.045 mmol) and halobenzene (0.045, 0.09, or 0.18 mmol) in chloroform (2 mL) and acetonitrile (1 mL). The **DPyA·1,4-C**_6_ **I**_2_ **F**_4_ cocrystals were obtained via solvent diffusion by adding **1,4-C**_6_ **I**_2_ **F**_4_ (0.09 mmol) dissolved in methanol (1 mL) into **DPyA** dissolved in chloroform (1.5 mL). Suitable single crystals for all samples formed via slow evaporation at room temperature, ca. 7 days after preparation. Phase purity was determined by the analysis of powder X-ray diffraction (PXRD) data.

### 2.2 X-ray Crystallography

SCXRD diffraction experiments were performed on a Rigaku XtaLAB Mini II diffractometer with a CCD area detector (λMoKα = 0.71073 Å, graphite monochromator). Standard data reduction and background correction were performed using the integrated CrysAlisPro package. Structural refinement and solution were performed with Olex2, SHELXL, and SHELXT.^34–36^ Crystallographic data and selected metrics for cocrystal structures are summarized in Tables S1–S5 (see the Supporting Information). PXRD data were collected on a Scintag XDS-2000 diffractometer using CuKα1 radiation (λ = 1.5418 Å). The samples were mounted and collected on glass slides typically in the range of 5**–**40° two-theta (scan type: step size: 0.02°, rate: 3 deg/min, continuous scan mode). The equipment was operated at 40 kV and 30 mA, and the data were collected at room temperature.

## 3 Results and Discussion

To evaluate the reliability of the synthon substitution approach for forming multicomponent solids of 9,10-diarylanthracene isosteres with halogenated coformers and to assess the resulting supramolecular architectures, we cocrystallized **DPA** and **DPyA** with **1,3,5-C**_6_ **I**_3_ **F**_3_, **1,2-C**_6_ **I**_2_ **F**_4_, and **1,4-C**_6_ **I**_2_ **F**_4_. SCXRD revealed the formation of cocrystals to be primarily supported by either [C–I···π] or [C–I···N] interactions (Table 1).

**Table 1:** Selected Metrics for Cocrystals with **DPA** and **DPyA** Isosteres^a^

o-crystal	*d*(I···N) (Å)	θ[π_cent_–N···I] (°)	*d*(I···π) (Å)	θ[π_cent_–C···I] (°)	ϕ(Ar_cent_–Ant_cent_) (°)	Mixed stack
(DPA)·2(1,2-C_6_I_2_F_4_)			3.745(2)	85.3(2)^b^	8.8(2)	1:2
(DPA)·(1,4-C_6_I_2_F_4_)^b^			3.550(2)	87.8(2)^c^	79.5(2)	1:1
(DPA)·4(1,3,5-C_6_I_3_F_3_)			3.665(4)	88.6(4)^d^	86.4(3)8	1:4
(DPyA)·(1,2-C_6_I_2_F_4_)	2.865(4)	167.7(2)			85.8(1)	1:1
(DPyA)·(1,4-C_6_I_2_F_4_)	2.9(1)	176.3(3)			88.3(3)	1:1
(DPyA)·2(1,3,5-C_6_I_3_F_3_)	2.852(4)	174.4(2)			89.4(2)	1:2

a π_cent_–C24···I.

b Coordinates from CCDC refcode: FONVAJ.

c π_cent_–C4···I.

d π_cent_–C11···I3.

### 3.1 DPA-Based Cocrystals

Cocrystal **DPA·1,3,5-C**_6_ **I**_3_ **F**_3_ crystallizes in the triclinic space group *P*-1 with an asymmetric unit that comprises one-half of **DPA** molecule and two **1,3,5-C**_6_ **I**_3_ **F**_3_ molecules. The **DPA** molecule exhibits a nearly orthogonal (86.4°) dihedral angle (ϕ) between the anthracenyl and phenyl rings, which is significantly more twisted than that of single-component **DPA** (65.3°).^37^ **DPA** molecules aggregate into layers along the *b*-axis via [C–H···π] contacts between the phenyl ring and the anthracene core, resembling the aggregation behavior reported in adamantane-based cocrystals with pyridines.^38^ In the **DPA·1,3,5–C**_6_ **I**_3_ **F**_3_ cocrystal, outer layers of **1,3,5-C**_6_ **I**_3_ **F**_3_ molecules (i.e., adjacent to **DPA** aggregates) are primarily supported by nearly orthogonal [C–I···π] contacts (86.6°) between the phenyl rings of **DPA** and aromatic rings of **1,3,5-C**_6_ **I**_3_ **F**_3_, as well as by [π···π] contacts (i.e., face-to-face π-stacking) with lateral anthracene (Figure 1a). Similar [C–I···π] contacts have been exploited for the construction of phosphorescent and pleochroic cocrystal solids.^11,39,40^ Additional inner layers of **1,3,5-C**_6_ **I**_3_ **F**_3_ units are supported by [I···I] halogen bonds, which assemble into trimers via a type II I_3_ synthon (Figure 1b).^41^ The extended packing reveals nontypical 1:4 mixed-stacks (**DPA**:**1,3,5-C**_6_ **I**_3_ **F**_3_) in the *ac*-plane (Figure 1c).^42,43^ A space-fill view of the extended packing shows **DPA·1,3,5-C**_6_ **I**_3_ **F**_3_ to arrange in close-packed, corrugated sheets in the *bc*-plane (Figure 1d).

**Figure 1:**
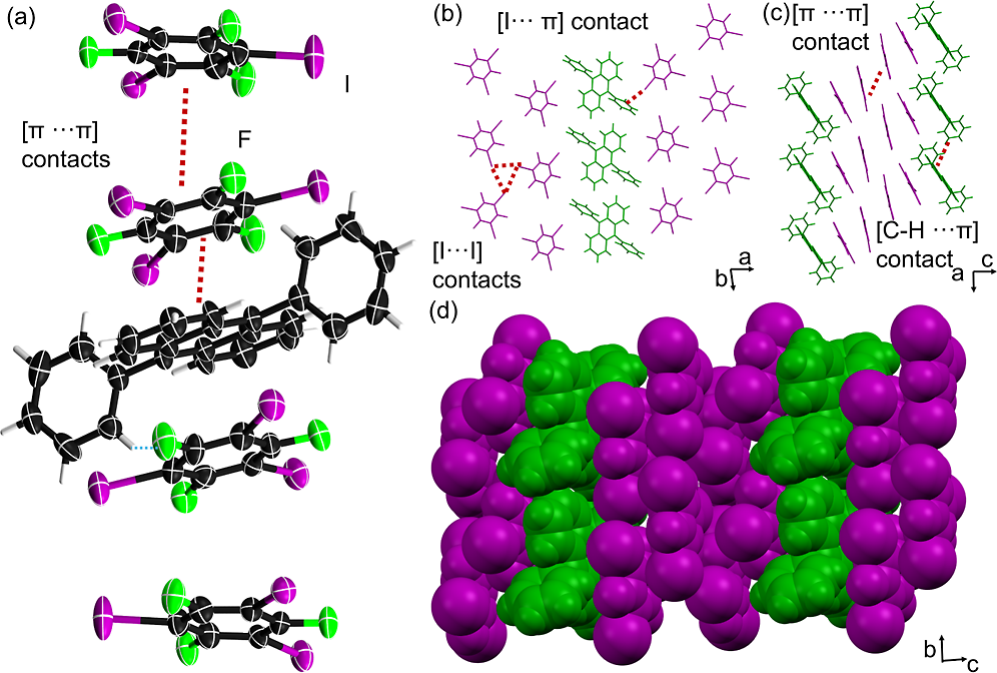
X-ray structure of **DPA·1,3,5–C**_6_ **I**_3_ **F**_3_: (a) 1:4 mixed-stack assembly, (b) [C–I···π] contacts and [I···I] contacts via a type II I_3_ synthon in the *ab*-plane, (c) mixed-stacks in the *ac* plane supported by [π···π] contacts, and (d) space-filling view of close-packed corrugated sheets in the *bc*-plane.

The components in **DPA·1,2-C**_6_ **I**_2_ **F**_4_ also crystallize in the triclinic space group *P*-1 with an asymmetric unit containing one-half of a **DPA** molecule and one **1,2-C**_6_ **I**_2_ **F**_4_ molecule. The **DPA** molecule displays a nearly orthogonal dihedral angle (88.8°) between the anthracenyl and phenyl rings, similar to that in **DPA·1,3,5-C**_6_ **I**_3_ **F**_3_ (Figure 2a). **DPA** units aggregate into zigzag-shaped layers along the *a*-axis via [C–H···π] contacts between a phenyl ring and a neighboring anthracene core. The **1,2-C**_6_ **I**_2_ **F**_4_ molecules engage in [π···π] contacts with anthracenyl rings and nearly orthogonal [C–I···π] contacts (87.8°) with phenyl rings, forming 1:2 mixed stacks in the *ac*-plane (Figure 2b), akin to donor–acceptor architectures in OSC materials.^43–45^ The **1,2-C**_6_ **I**_2_ **F**_4_ units adopt a head-to-tail geometry, as observed in cocrystals with pyrene.^39^ The overall structure forms close-packed, corrugated sheets parallel to the *bc*-plane (Figure 2c), contrasting the 1:4 mixed stacks observed in the **DPA·1,3,5–C**_6_ **I**_3_ **F**_3_ cocrystal.

**Figure 2:**
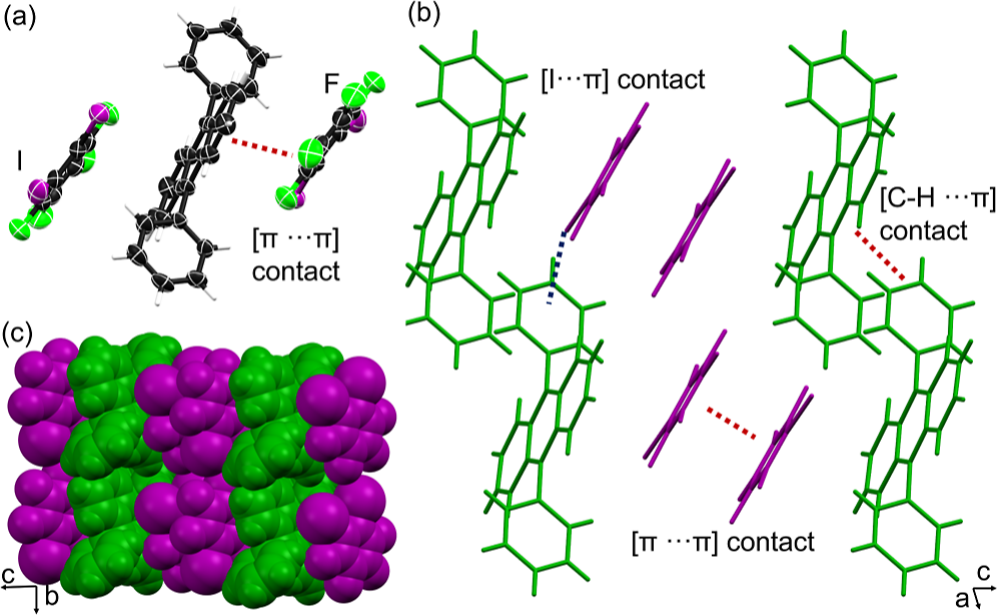
X-ray structure of **DPA·1,2-C**_6_ **I**_2_ **F**_4_: (a) three-component assembly, (b) 2:1 mixed stacks supported by [π···π] contacts and [C–I···π] contacts, and (c) space-filling view of corrugated sheets along the *b*-axis.

The components in the **DPA·1,4-C**_6_ **I**_2_ **F**_4_ cocrystal^20^ crystallize in the triclinic space group *P*-1 with an asymmetric unit comprising one **DPA** molecule and one **1,4-C**_6_ **I**_2_ **F**_4_ molecule. The **DPA** molecule is less twisted (79.5°) than in the **DPA·1,3,5-C**_6_ **I**_3_ **F**_3_ and **DPA·1,2-C**_6_ **I**_2_ **F**_4_ cocrystals. In the solid, **DPA** molecules form layers connected by [C–H···π] contacts and generate 1:1 mixed stacks with intercalated **1,4-C**_6_ **I**_2_ **F**_4_ units through [π···π] contacts, further stabilized by nearly orthogonal [C–I···π] contacts (88.6°) with phenyl rings (Figure S1). Cocrystals of **1,4-C**_6_ **I**_2_ **F**_4_ with other PAHs (e.g., phenanthrene, chrysene, pyrene) have shown the prevalence of [C–I···π] contacts to support the formation of multicomponent solids.^46^

### 3.2 DPyA-Based Cocrystals

To demonstrate the use of [C–I···N] halogen bonds as reliable supramolecular synthon substitutes for [C–I···π] interactions in a molecular isostere of **DPA**, the pyridyl-containing derivative **DPyA** was cocrystallized with the same series of halogenated coformers.

The components in **DPyA·1,3,5-C**_6_ **I**_3_ **F**_3_ crystallize in the triclinic space group *P*-1 with an asymmetric unit containing one-half of **DPyA** molecule and one **1,3,5-C**_6_ **I**_3_ **F**_3_ molecule interacting through a [C–I···N] halogen bond. The halogen bond distance (2.852 Å) is comparable to those observed in pyridine cocrystals and nearly linear (174.4°), which is in agreement with a directional σ-hole interaction (Figure 3a).^47,48^ The **DPyA** molecule exhibits a dihedral angle of 89.4° between the anthracenyl and pyridyl rings, which is the most twisted conformation among the series and significantly larger than that of single-component **DPyA** (71.9°).^49^ **DPyA** molecules arrange into zigzag-shaped layers along the *a*-axis via [C–H···π] contacts. The layers sandwich two **1,3,5-C**_6_ **I**_3_ **F**_3_ molecules through [π···π] contacts in the *ac*-plane (Figure 3b). Additional type II [I···I] halogen bonds further support the aggregation of **1,3,5-C**_6_ **I**_3_ **F**_3_ molecules.^50–52^ The extended packing forms close-packed corrugated sheets in the *bc*-plane (Figure 3c). Notably, [C–I···π] contacts are absent in the **DPyA·1,3,5-C**_6_ **I**_3_ **F**_3_ cocrystal, which can be rationalized by the more localized electron density at the pyridyl nitrogen atoms in **DPyA** relative to the more diffuse π systems of **DPA**. This localization favors the formation of directional [C–I···N] halogen bonds over competing [C–I···π] interactions, consistent with electrostatic potential (ESP) maps (Figure S3).^53^

**Figure 3:**
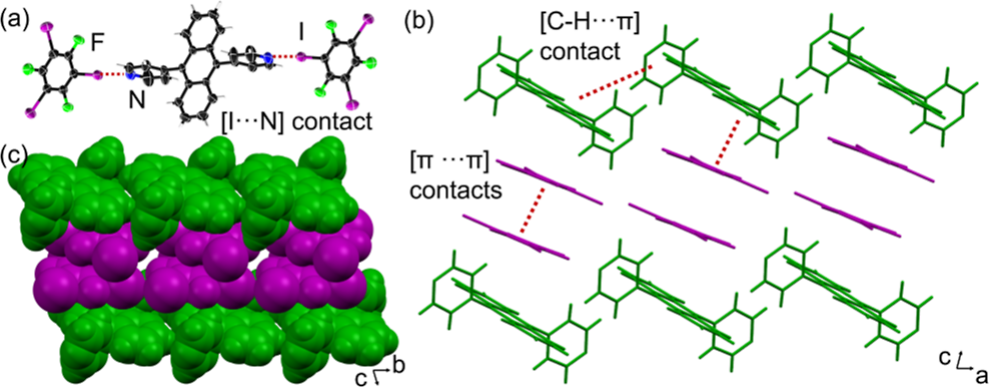
X-ray structure of **DPyA·1,3,5-C**_6_ **I**_3_ **F**_3_: (a) three-component assembly supported by [C–I···N] contacts, (b) 1:2 mixed stacks supported by [π···π] contacts in the *ac* plane, and (c) space-filling view of corrugated sheets along the *b*-axis.

The components in the **DPyA·1,2-C**_6_ **I**_2_ **F**_4_ cocrystal crystallize in the monoclinic space group *C*2/*c* with an asymmetric unit that contains one-half of a **DPyA** molecule and one-half of a **1,2-C**_6_ **I**_2_ **F**_4_ unit. The components interact via a [C–I···N] halogen bond (2.865 Å), comparable to that of **DPyA·1,3,5-C**_6_ **I**_3_ **F**_3_ (Figure 4a). When grown by symmetry, alternating **DPyA** and **1,2-C**_6_ **I**_2_ **F**_4_ molecules assemble into zigzag chains sustained by [C–I···N] interactions, reminiscent of the supramolecular structure reported for a cocrystal with **1,2-C**_6_ **I**_2_ **F**_4_ and 1,4-diazabicyclo[2.2.2]­octane.^54^ Aggregates of **DPyA** molecules supported by [C–H···π] contacts sandwich a single layer of **1,2-C**_6_ **I**_2_ **F**_4_ units in the *ac*-plane. The interaction between **DPyA** and coformer molecules occurs via [π···π] contacts (Figure 4b). No [C–I···π] contacts are observed in **DPyA·1,2-C**_6_ **I**_2_ **F**_4_. A space-filling view shows the components in **DPyA·1,2-C**_6_ **I**_2_ **F**_4_ to arrange in a close-packed architecture in the *bc*-plane (Figure 4c).

**Figure 4:**
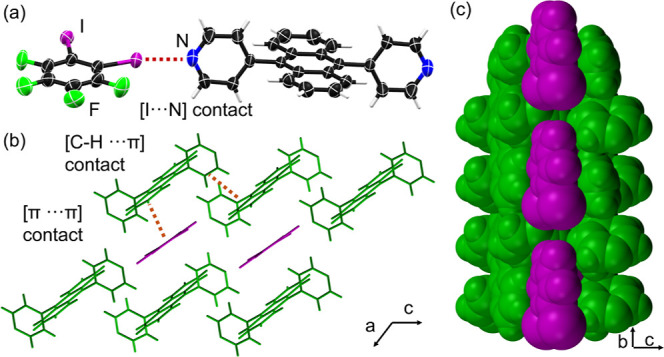
X-ray structure of **DPyA·1,2-C**_6_ **I**_2_ **F**_4_: (a) three-component assembly supported by [C–I···N] contacts, (b) 1:1 mixed stacks supported by [π···π] contacts in the *ac* plane, and (c) space-filling view of corrugated sheets along the *b*-axis.

Cocrystal **DPyA·1,4C**_6_ **I**_2_ **F**_4_ crystallizes in the monoclinic space group *C*2/*m* with an asymmetric unit comprising one-half of a **DPyA** molecule and one-half of a **1,4-C**_6_ **I**_2_ **F**_4_ molecule interacting via [C–I···N] halogen bonds (Figure 5a). **DPyA·1,4-C**_6_ **I**_2_ **F**_4_ forms linear chains of alternating **DPyA** and **1,4-C**_6_ **I**_2_ **F**_4_ units sustained by directional [C–I···N] contacts, similar to those observed in cocrystals of 4,4′-azopyridine and 1,2-bis­(4-pyridyl)­ethene with **1,4-C**_6_ **I**_2_ **F**_4_.^55^ Within the chains, the components show two disordered positions of the anthracene core, pyridine, and **1,4-C**_6_ **I**_2_ **F**_4_ rings, maintaining near-orthogonal dihedral angles (88.3°). **DPyA** chains further organize into layers sustained by [C–H···π] and van der Waals [H···H] contacts that sandwich single layers of **1,4-C**_6_ **I**_2_ **F**_4_ in the *bc*-plane, forming 1:1 mixed stacks (Figure 5b–d). The overall structure is close-packed and without voids (Figure 5e). The versatility of **1,4-C**_6_ **I**_2_ **F**_4_ as a coformer with various halogen-bond acceptors is well-established.^56^ We note the identification of an iodine atom next to the ipso-carbon of the pyridine ring is attributed to trace (4% occupancy) 9,10-diiodoanthracene impurity, generating a mixed cocrystal.^57^

**Figure 5:**
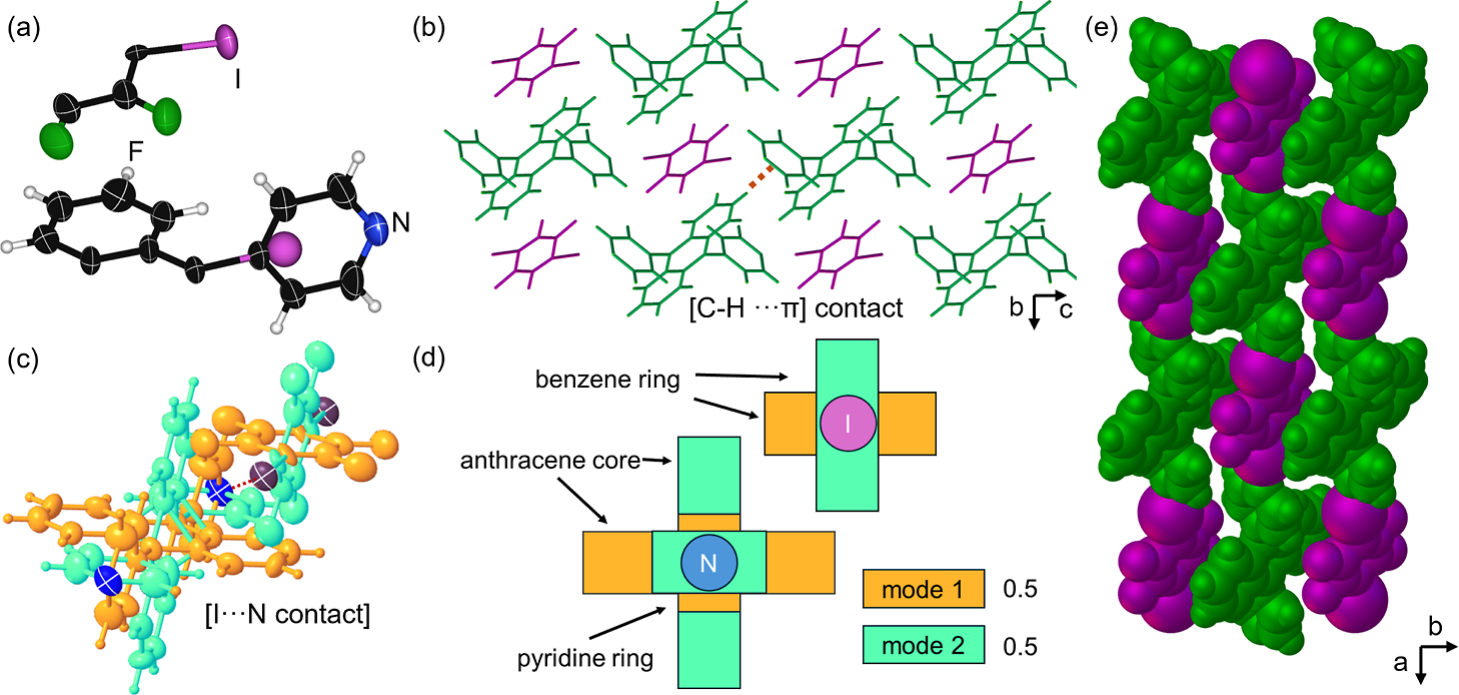
X-ray structure of **DPyA·1,4-C**_6_ **I**_2_ **F**_4_: (a) asymmetric unit containing one-half-molecule of **1,4-C**_6_ **I**_2_ **F**_4_ and a half of a **DPyA** unit that includes a trace iodine atom from 9,10-diiodoanthracene. We note the C–H and C–I bonds connected to the ipso-carbon of the anthracene closely overlap. (b) [C–H···π] contacts between **DPyA** units facilitate the inclusion of layers of **1,4-C**_6_ **I**_2_ **F**_4_ in the *bc*-plane. (c) Pseudodirectional chains via [I···N] contacts along the *a*-axis. (d) Schematics of the observed disordered. (e) Space-filling view of the **DPyA·1,4-C**_6_ **I**_2_ **F**_4_ assembly.

### 3.3 Rationale toward Supramolecular Synthon Substitution

Hirshfeld surface analyses were performed using CrystalExplorer17 ^58^ to gain deeper insight into the intermolecular interactions governing the synthesized cocrystals (Figure 6). For each pair of isosteric cocrystals (i.e., **DPA·1,2-C**_6_ **I**_2_ **F**_4_ vs **DPyA·1,2-C**_6_ **I**_2_ **F**_4_, **DPA·1,4-C**_6_ **I**_2_ **F**_4_ vs **DPyA·1,4-C**_6_ **I**_2_ **F**_4_, and **DPA·1,3,5-C**_6_ **I**_3_ **F**_3_ vs **DPyA·1,3,5-C**_6_ **I**_3_ **F**_3_), the N → C replacement in the aryl handle leads to an increased contribution of [C···I] contacts, corresponding to the replacement of N···I interactions. In **DPA**-based systems exhibiting mixed 1:2 and 1:4 stacks (i.e., **DPA·1,2-C**_6_ **I**_2_ **F**_4_ and **DPA·1,3,5-C**_6_ **I**_3_ **F**_3_), additional [F···I] contacts are observed, which is consistent with the higher inclusion of halobenzene coformers. Variations in the proportions of [C···H] contacts across the isosteric pairs reflect differences in molecular aggregation and proximity between neighboring **DPA**–**DPA** and **DPyA**–**DPyA** molecules mediated by [C–H···π] contacts. The largest difference occurs between structures **DPA·1,2-C**_6_ **I**_2_ **F**_4_ and **DPyA·1,2-C**_6_ **I**_2_ **F**_4_, corresponding to a ca. 0.4 Å difference between the aryl hydrogen atom and the centroid of the anthracene side ring. Notably, the aggregation of three **1,3,5-C**_6_ **I**_3_ **F**_3_ units via the type II I_3_ synthon in **DPA·1,3,5-C**_6_ **I**_3_ **F**_3_ contributes 4.8% of I···I contacts, which is not significant in other cocrystals, and accounts for the highest coformer inclusion ratio (1:4) in the series. Another relevant difference is the higher contribution of [H···H] contacts in **DPyA·1,4-C**_6_ **I**_2_ **F**_4_ than that of **DPA·1,4-C**_6_ **I**_2_ **F**_4_, which could be attributed to the close-packed interactions between chains of **DPyA** units. The chains of **DPyA** molecules in **DPyA·1,4-C**_6_ **I**_2_ **F**_4_ are disordered in over two positions, generating increased [H···H] contacts with four adjacent chains (Figure 5c). This motif is absent in **DPA·1,4-C**_6_ **I**_2_ **F**_4_ (Figure S1).

**Figure 6:**
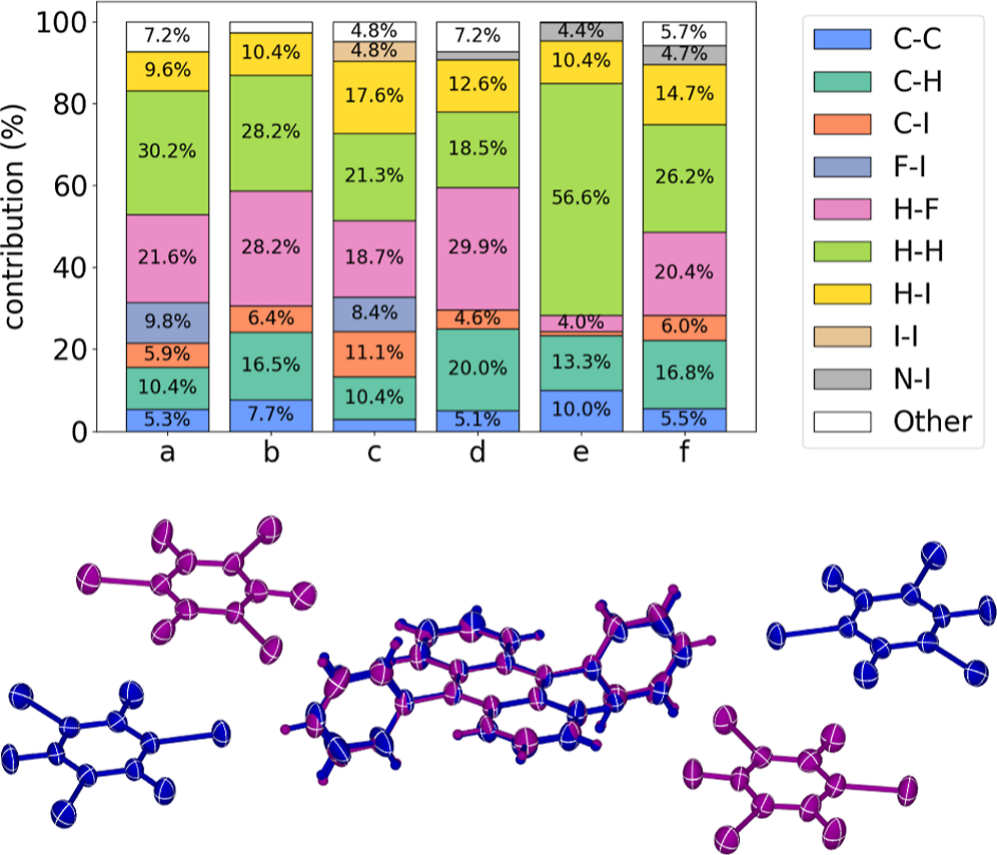
(Top) Hirshfeld surface analyses for (a) **DPA·1,2-C**_6_ **I**_2_ **F**_4_, (b) **DPA·1,4-C**_6_ **I**_2_ **F**_4_ (c) **DPA·1,3,5-C**_6_ **I**_3_ **F**_3_, (d) **DPyA·1,2-C**_6_ **I**_2_ **F**_4_, (e) **DPyA·1,4-C**_6_ **I**_2_ **F**_4_, and (f) **DPyA·1,3,5-C**_6_ **I**_3_ **F**_3_. (Bottom) Overlay of crystal structures of **DPA·1,3,5-C**_6_ **I**_3_ **F**_3_ and **DPyA·1,3,5-C**_6_ **I**_3_ **F**_3_ cocrystals, showing the preservation of the molecular conformation and positioning variability of coformers.

Interaction energy analysis obtained from CrystalExplorer calculations (Table S1) supports the results. In the **DPA** cocrystals, the most stabilizing molecular pairs are associated with arrangements that feature diffuse [C–I···π] contacts (ca. – 14 to – 20 kJ mol^–1^), supplemented by additional coformer–coformer interactions enabled by [π···π], [π···F], and [I···I] contacts. In contrast, the **DPyA** analogues are driven by **DPyA**–coformer molecular pairs of comparable interaction strength (ca. – 19 kJ mol^–1^), which are associated with localized and highly directional [C–I···N] halogen bonds. These interactions efficiently stabilize the lattice, lock in the supramolecular architecture, and favor lower coformer inclusion. ESP maps provide further support for this observation, showing the minima (i.e., most electron-rich) to be localized at the pyridyl nitrogen atoms in **DPyA**, whereas more spatially diffused minima are distributed over the π-systems of **DPA** (Figure S3). The localization of electron density at the nitrogen atom provides a rationale for the preferential formation of [C–I···N] halogen bonds and the reduced prevalence of competing [C–I···π] contacts.

Despite these differences, the molecular conformations of both isosteres remain preserved across all cocrystals. Geometry-optimized structures indicate that, while the anthracene cores are rigid, the pendant phenyl and pyridyl rings retain limited but meaningful rotational freedom, adopting larger dihedral angles in the gas-phase optimized structures (ca. 90°) compared to the solid-state conformations of single component **DPA** and **DPyA** (ca. 67–72°),^49,59^ enabling interaction with coformers in cocrystals (ca. 80–90°) without perturbing the chromophore core (Figure S2). In **DPyA** solids, coformers adopt near-linear arrangements enforced by [C–I···N] halogen bonds, whereas in **DPA** analogues, they engage in less constrained [C–I···π] contacts. Collectively, these results demonstrate that supramolecular synthon substitution provides a reliable and predictive strategy for the assembly of cocrystals of similar OSC isosteres.

### 3.4 Photophysical Properties

The preservation of the molecular conformations of the OSCs prompted us to investigate the photophysical properties of the synthesized cocrystals. Fluorescence spectra of suspensions of single crystals in heptane were recorded on a PTI QuantaMaster 400 fluorometer for the three pairs of isosteric cocrystals (Figure 7). The emission profiles of all **DPA**- and **DPyA**-based cocrystals closely resemble those of the parent compounds, indicating the intrinsic excited-state characteristics of the anthracene core are largely retained (Figure S5).^60,61^ The spectral consistency is attributed to the preservation of the twisted geometry between the anthracene core and the aryl substituents (phenyl or pyridyl), as well as the presence of [C–H···π] contacts across all six cocrystal structures.^62^ The excitation spectra match those of pure **DPA** and **DPyA** within the 300–400 nm region, exhibiting only minor deviations in the near-UV region. The subtle differences are likely associated with distinct halogen–π and halogen–nitrogen interaction geometries in the cocrystals, which may slightly modulate the relative oscillator strengths of high-energy transitions through the iodine-induced heavy-atom effect.^63,64^ Overall, the results demonstrate that supramolecular synthon substitution enables the formation of isosteric cocrystals that maintain the photophysical integrity of the parent OSC chromophore while introducing controlled structural diversity through noncovalent interactions.

**Figure 7:**
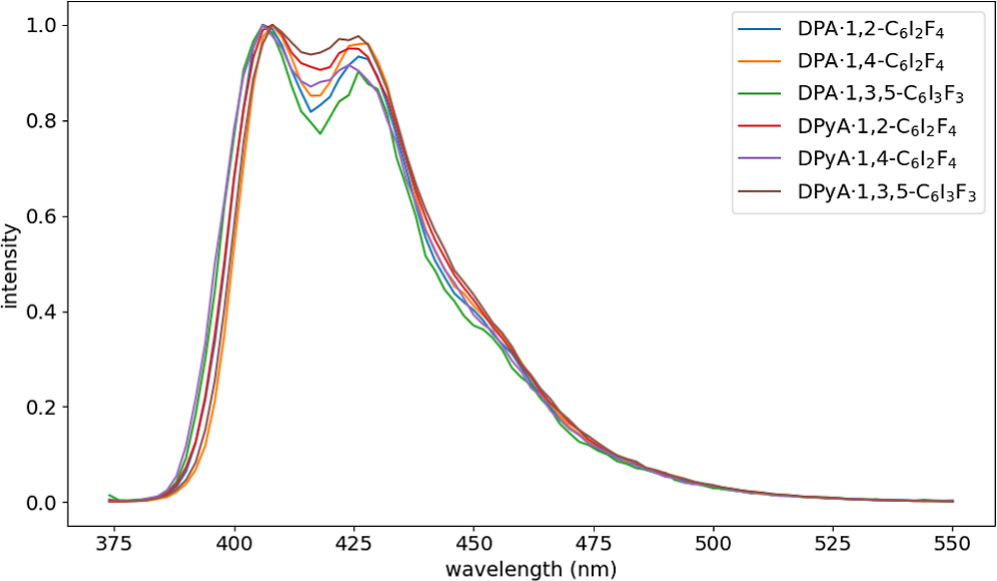
Emission spectra for cocrystal systems (λ_ex_ = 372 nm for **DPyA·1,4-C**_6_ **I**_2_ **F**_4_ and λ_ex_ = 370 nm for the rest of the systems).

## 4 Conclusion

In summary, our study has demonstrated supramolecular synthon substitution to be a reliable tool for the design of multicomponent solids of 9,10-diarylanthracene isosteres with halogenated coformers **1,3,5-C**_6_ **I**_3_ **F**_3_, **1,2-C**_6_ **I**_2_ **F**_4_, and **1,4-C**_6_ **I**_2_ **F**_4_. Specifically, cocrystals of **DPA** and **DPyA** exhibit a consistent replacement of [C–I···π] contacts in **DPA** solids by [C–I···N] halogen bonds in **DPyA** isosteric analogues. Changes in coformer geometry and synthon type result in mixed π-stacked assemblies with variable stoichiometries (i.e., 1:1, 1:2, and 1:4 OSC/coformer ratios), showing the versatility of halogen bond contacts in directing solid-state architectures. These stoichiometric variations can be rationalized by differences in synthon efficiency, with localized [C–I···N] halogen bonds stabilizing **DPyA**-based cocrystals with fewer coformer molecules, whereas more diffuse [C–I···π] interactions in **DPA** analogues are complemented by additional coformer–coformer contacts. Notably, despite structural differences, the molecular conformations and photophysical properties of **DPA** and **DPyA** remain largely preserved across all of the solids. The results demonstrate the potential of synthon substitution as a predictive design element in crystal engineering and provide a platform for the diversification of properties and supramolecular architectures of well-established OSCs and related PAHs.

## Supplementary Material



## Notes

Experimental information, molecular modeling data, excitation spectra, PXRD patterns, and additional SCXRD data X-ray crystallographic information files (CIF) are available for **DPA·1,3,5-C**_6_ **I**_3_ **F**_3_, **DPA·1,2-C**_6_ **I**_2_ **F**_4_, **DPyA·1,3,5-C**_6_ **I**_3_ **F**_3_, **DPyA·1,2-C**_6_ **I**_2_ **F**_4_, **DPyA·1,4-C**_6_ **I**_2_ **F**_4_ (CCDC 2492528–2492532) (PDF)

## References

[1] Allard S., Forster M., Souharce B., Thiem H., Scherf U. (2008). Organic Semiconductors
for Solution-Processable Field-Effect Transistors (OFETs). Angew. Chem., Int. Ed..

[2] Khasbaatar A., Xu Z., Lee J.-H., Campillo-Alvarado G., Hwang C., Onusaitis B. N., Diao Y. (2023). From Solution to Thin Film: Molecular Assembly of π-Conjugated
Systems and Impact on (Opto)­electronic Properties. Chem. Rev..

[3] Wei C., Li L., Zheng Y., Wang L., Ma J., Xu M., Lin J., Xie L., Naumov P., Ding X., Feng Q., Huang W. (2024). Flexible Molecular
Crystals for Optoelectronic Applications. Chem.
Soc. Rev..

[4] Karothu D. P., Dushaq G., Ahmed E., Catalano L., Rasras M., Naumov P. (2021). Multifunctional Deformable
Organic Semiconductor Single
Crystals. Angew. Chem., Int. Ed..

[5] Jiang H., Hu W. (2020). Towards High-Performance Organic
Field-Effect Transistors: Influence
of Molecular Packing of Organic Semiconductors. Angew. Chem., Int. Ed..

[6] Zhang X., Dong H., Hu W. (2018). Organic Semiconductor Single Crystals
for Electronics and Photonics. Adv. Mater..

[7] Jiang L., Dong H., Hu W. (2010). Organic Single Crystal Field-Effect
Transistors: Advances and Perspectives. J. Mater.
Chem..

[8] Reese C., Bao Z. (2007). Organic Single-Crystal
Field-Effect Transistors. Mater. Today.

[9] Colatrella A., Bondarenko I., Liu R. J., Bernhardt M., Chang T., Chen Y.-S., Campillo-Alvarado G. (2025). Temperature-driven
molecular dynamics initiate thermochromism and luminescence modulation
in an anthracene-based organic semiconductor crystal. J. Mater. Chem. C.

[10] Li X., Pan F., Sun C., Zhang M., Wang Z., Du J., Wang J., Xiao M., Xue L., Zhang Z.-G., Zhang C., Liu F., Li Y. (2019). Simplified Synthetic
Routes for Low Cost and High Photovoltaic Performance *n*-Type Organic Semiconductor Acceptors. Nat.
Commun..

[11] Vainauskas J., Borchers T. H., Arhangelskis M., McCormick McPherson L. J., Spilfogel T. S., Hamzehpoor E., Topić F., Coles S. J., Perepichka D. F., Barrett C. J., Friščić T. (2023). Halogen bonding
with carbon: directional assembly of non-derivatised aromatic carbon
systems into robust supramolecular ladder architectures. Chem. Sci..

[12] Campillo-Alvarado G., Bernhardt M., Davies D. W., Soares J. A. N. T., Woods T. J., Diao Y. (2021). Modulation
of π-Stacking Modes
and Photophysical Properties of an Organic Semiconductor through Isosteric
Cocrystallization. J. Chem. Phys..

[13] Hinoue T., Shigenoi Y., Sugino M., Mizobe Y., Hisaki I., Miyata M., Tohnai N. (2012). Regulation of π-Stacked Anthracene
Arrangement for Fluorescence Modulation of Organic Solid from Monomer
to Excited Oligomer Emission. Chem.—Eur.
J..

[14] Sugino M., Hatanaka K., Araki Y., Hisaki I., Miyata M., Tohnai N. (2014). Amphiphilic Inclusion
Spaces for Various Guests and
Regulation of Fluorescence Intensity of 1,8-Bis­(4-aminophenyl)­anthracene
Crystals. Chem.—Eur. J..

[15] Liu Y., Li A., Xu S., Xu W., Liu Y., Tian W., Xu B. (2020). Crystal Engineering of Organic Luminogens
with Efficient Room-Temperature
Phosphorescence by Halogen Bonding. Angew. Chem.,
Int. Ed..

[16] Sokolov A. N., Friščić T., MacGillivray L. R. (2006). Instant
Cocrystallization via Halogen-Bonding-Driven Self-Assembly. J. Am. Chem. Soc..

[17] Hutchins K.
M. (2018). Functional
Materials Based on Molecules with Hydrogen-Bonding Ability: Applications
to Drug Co-crystals and Polymer Complexes. R.
Soc. Open Sci..

[18] Sonina A. A., Cheshkina D. S., Kazantsev M. S. (2023). Additive-Assisted
Crystallization
of 9,10-Diphenylanthracene. Crystals.

[19] Bosch E., Reinheimer E. W., Unruh D. K., Groeneman R. H. (2023). Co-crystal
Sustained by π-Type Halogen-Bonding Interactions between 1,4-Diiodoperchlorobenzene
and Naphthalene. Acta Crystallogr., Sect. C:
Struct. Chem..

[20] Fan G., Yan D. (2016). Two-Component Orderly
Molecular Hybrids of Diphenylanthracene: Modulation
of Solid-State Aggregation toward Tunable Photophysical Properties
and Highly Enhanced Electrochemiluminescence. Adv. Opt. Mater..

[21] Yang B., Xiao J., Wong J. I., Guo J., Wu Y., Ong L., Lao L. L., Boey F., Zhang H., Yang H. Y. (2011). Shape-Controlled Micro/Nanostructures
of 9,10-Diphenylanthracene
(DPA) and Their Application in Light-Emitting Devices. J. Phys. Chem. C.

[22] Vinyard D.
J., Su S., Richter M. M. (2008). Electrogenerated Chemiluminescence of 9,10-Diphenylanthracene,
Rubrene, and Anthracene in Fluorinated Aromatic Solvents. J. Phys. Chem. A.

[23] Zhu T., Liu X., Qin J., Hu Q., Ning J. (2019). Studies on the Luminescence
and Scintillation Properties of 9,10-Diphenylanthracene Crystals Grown
by Two Methods. Cryst. Growth Des..

[24] Haubitz T., Fudickar W., Linker T., Kumke M. U. (2020). pH-Sensitive
Fluorescence
Switching of Pyridylanthracenes: The Effect of the Isomeric Pattern. J. Phys. Chem. A.

[25] Desiraju G. R. (1995). Supramolecular
Synthons in Crystal Engineering—A New Organic Synthesis. Angew. Chem., Int. Ed. Engl..

[26] Aakeröy C. B., Panikkattu S., Chopade P. D., Desper J. (2013). Competing Hydrogen-Bond
and Halogen-Bond Donors in Crystal Engineering. CrystEngComm.

[27] Quentin J., MacGillivray L. R. (2020). Halogen
versus Hydrogen Bonding in Binary Cocrystals:
Novel Conformation of a Coformer with [2 + 2] Photoreactivity of Criss-Crossed
C = C Bonds. ChemPhysChem.

[28] Amonov A., Scheiner S. (2025). Replacement of Hydrogen by Halogen Bonds within Nucleic
Acid Base Pairs. New J. Chem..

[29] Shen Q. J., Pang X., Zhao X. R., Gao H. Y., Sun H.-L., Jin W. J. (2012). Phosphorescent cocrystals constructed by 1, 4-diiodotetrafluorobenzene
and polyaromatic hydrocarbons based on C–I π halogen
bonding and other assisting weak interactions. CrystEngComm.

[30] Bedeković N., Stilinović V., Friščić T., Cinčić D. (2018). Comparison
of isomeric meta-and para-diiodotetrafluorobenzene
as halogen bond donors in crystal engineering. New J. Chem..

[31] Torubaev Y. V., Skabitsky I. V. (2024). Novel Supramolecular
Heterosynthons [C–I···
N N] and [C–I··· π (C≡C)]. Cryst. Growth Des..

[32] Hutchins K. M., Unruh D. K., Carpenter D. D., Groeneman R. H. (2018). Thermal
expansion along one-dimensional chains and two-dimensional sheets
within co-crystals based on halogen or hydrogen bonds. CrystEngComm.

[33] Liu Y., Li A., Xu S., Xu W., Liu Y., Tian W., Xu B. (2020). Reversible Luminescent
Switching in an Organic Cocrystal: Multi-Stimuli-Induced
Crystal-To-Crystal Phase Transformation. Angew.
Chem., Int. Ed..

[34] Dolomanov O. V., Bourhis L. J., Gildea R. J., Howard J. A., Puschmann H. (2009). OLEX2: a complete
structure solution, refinement and analysis program. J. Appl. Crystallogr..

[35] Sheldrick G. M. (2015). Crystal
structure refinement with SHELXL. Acta Crystallogr.,
Sect. C: Struct. Chem..

[36] Sheldrick G. M. (2015). SHELXT–Integrated
space-group and crystal-structure determination. Acta Crystallogr., Sect. A: Found. Crystallogr..

[37] Fujiwara Y., Ozawa R., Onuma D., Suzuki K., Yoza K., Kobayashi K. (2013). Double alkylene-strapped diphenylanthracene
as a photostable
and intense solid-state blue-emitting material. J. Org. Chem..

[38] Bicknell J., Agarwal S. A., Petersen K. J., Loya J. D., Lutz N., Sittinger P. M., Teat S. J., Settineri N. S., Campillo-Alvarado G. (2024). Engineering
Lipophilic Aggregation of Adapalene and
Adamantane-Based Cocrystals via van der Waals Forces and Hydrogen
Bonding. Cryst. Growth Des..

[39] Shen Q. J., Wei H. Q., Zou W. S., Sun H. L., Jin W. J. (2012). Cocrystals
assembled by pyrene and 1, 2-or 1, 4-diiodotetrafluorobenzenes and
their phosphorescent behaviors modulated by local molecular environment. CrystEngComm.

[40] Vainauskas J., Topić F., Bushuyev O. S., Barrett C. J., Friščić T. (2020). Halogen bonding
to the azulene π-system: cocrystal design of pleochroism. Chem. Commun..

[41] Li B., Zang S.-Q., Wang L.-Y., Mak T. C. (2016). Halogen bonding:
A powerful, emerging tool for constructing high-dimensional metal-containing
supramolecular networks. Coord. Chem. Rev..

[42] Wang Y., Zhu W., Dong H., Zhang X., Li R., Hu W. (2019). Organic cocrystals:
new strategy for molecular collaborative innovation. Mol.-Scale Electron.: Curr. Status Perspect..

[43] Sun L., Wang Y., Yang F., Zhang X., Hu W. (2019). Cocrystal
engineering: a collaborative strategy toward functional materials. Adv. Mater..

[44] Mathur C., Gupta R., Bansal R. K. (2024). Organic
Donor-Acceptor Complexes
As Potential Semiconducting Materials. Chem.—Eur.
J..

[45] Ray K. K., Campillo-Alvarado G., Morales-Rojas H., Hopfl H., MacGillivray L. R., Tivanski A. V. (2020). Semiconductor cocrystals
based on boron: generated
electrical response with π-rich aromatic molecules. Cryst. Growth Des..

[46] Abe A., Goushi K., Mamada M., Adachi C. (2024). Organic binary and
ternary cocrystal engineering based on halogen bonding aimed at room-temperature
phosphorescence. Adv. Mater..

[47] Cavallo G., Metrangolo P., Milani R., Pilati T., Priimagi A., Resnati G., Terraneo G. (2016). The halogen bond. Chem. Rev..

[48] Côté M., Ovens J. S., Bryce D. L. (2023). Anticooperativity
and Competition
in Some Cocrystals Featuring Iodine-Nitrogen Halogen Bonds. Chem.—Asian J..

[49] Cui X., Khlobystov A. N., Chen X., Marsh D. H., Blake A. J., Lewis W., Champness N. R., Roberts C. J., Schröder M. (2009). Dynamic Equilibria
in Solvent-Mediated Anion, Cation and Ligand Exchange in Transition-Metal
Coordination Polymers: Solid-State Transfer or Recrystallisation?. Chem.—Eur. J..

[50] Campillo-Alvarado G., Li C., Swenson D. C., MacGillivray L. R. (2019). Application of Long-Range Synthon
Aufbau Modules Based on Trihalophenols To Direct Reactivity in Binary
Cocrystals: Orthogonal Hydrogen Bonding and π–π
Contact Driven Self-Assembly with Single-Crystal Reactivity. Cryst. Growth Des..

[51] Mukherjee A., Tothadi S., Desiraju G. R. (2014). Halogen bonds in crystal engineering:
like hydrogen bonds yet different. Acc. Chem.
Res..

[52] Lutz N., Bicknell J., Loya J. D., Reinheimer E. W., Campillo-Alvarado G. (2024). Channel confinement and separation
properties in an
adaptive supramolecular framework using an adamantane tecton. CrystEngComm.

[53] Gao H., Zhao X., Wang H., Pang X., Jin W. (2013). Cocrystal
Assembled by 1, 2-Diiodotetrafluorobenzene and Acridine via C- I···
N Halogen Bond and π-hole···F Bonds. Chin. J. Chem..

[54] Arhangelskis M., Topić F., Hindle P., Tran R., Morris A. J., Cinčić D., Friščić T. (2020). Mechanochemical
reactions of cocrystals: comparing theory with experiment in the making
and breaking of halogen bonds in the solid state. Chem. Commun..

[55] Ravat P., SeethaLekshmi S., Biswas S. N., Nandy P., Varughese S. (2015). Equivalence
of ethylene and azo-bridges in the modular design of molecular complexes:
role of weak interactions. Cryst. Growth Des..

[56] Ding X.-H., Chang Y.-Z., Ou C.-J., Lin J.-Y., Xie L.-H., Huang W. (2020). Halogen bonding in the co-crystallization
of potentially ditopic
diiodotetrafluorobenzene: a powerful tool for constructing multicomponent
supramolecular assemblies. Natl. Sci. Rev..

[57] George G. C., Ma L., Gaffney J. R., Brooks R. K., Unruh D. K., Groeneman R. H., Hutchins K. M. (2025). Tuning thermomechanical
properties of hydrogen-bonded
materials by using a mixed cocrystal approach. J. Mater. Chem. C.

[58] Spackman P. R., Turner M. J., McKinnon J. J., Wolff S. K., Grimwood D. J., Jayatilaka D., Spackman M. A. (2021). CrystalExplorer: a program for Hirshfeld
surface analysis, visualization and quantitative analysis of molecular
crystals. J. Appl. Crystallogr..

[59] Langer V., Becker H.-D. (1992). Crystal structure
of 9, 10-diphenylanthracene,(C6H5)­(C14H8)­(C6H5). Z. Kristallogr..

[60] Taniguchi M., Lindsey J. S. (2018). Database of Absorption
and Fluorescence Spectra of
> 300 Common Compounds for use in PhotochemCAD. Photochem. Photobiol..

[61] Jia J., Zhang T., Lu Y., Wu X., Song Y. (2026). Dramatically
enhanced broadband reverse saturable absorption of anthracene derivatives:
Regulatory effect of π-bridge in two-branched molecules. J. Photochem. Photobiol., A.

[62] Feng Q., Wang M., Dong B., He J., Xu C. (2013). Regulation
of Arrangements of Pyrene Fluorophores via Solvates and Cocrystals
for Fluorescence Modulation. Cryst. Growth &
Design.

[63] Adeniyi E., Rosokha S. V. (2025). Strengths, nature
and spectral implications of the
σ–n *vs*. σ–π halogen
bonding between diiodine and aromatic amines: a computational study. Phys. Chem. Chem. Phys..

[64] Ansari R., Hashemi D., Kieffer J. (2021). The role of
halogen bonding in metal
free phosphors. Phys. Chem. Chem. Phys..

