# Supplementary material for: Synthon Substitution via C–I···π and C–I···N Halogen Bonds in Cocrystals of Anthracene-Based Organic Semiconductor Isosteres
Source: Cryst Growth Des. 2026 Jan 13;26(3):1343–50. doi: 10.1021/acs.cgd.5c01513 (PMC12879540; doi:10.1021/acs.cgd.5c01513)
Supplement: Supplementary file 1 [file cg5c01513_si_001.pdf]

Supporting Information (SI)

Synthon Substitution via C–I$\cdots\pi$ and C–I$\cdots$N Halogen Bonds in Co-crystals of Anthracene-Based Organic Semiconductor Isosteres

Ivan Bondarenko,† Shivani Ahuja,† Brian O. Patrick,‡ and Gonzalo Campillo-Alvarado*,†

†*Department of Chemistry, Reed College, Portland, Oregon 97202-8199, United States*

‡*Department of Chemistry, University of British Columbia, 2036 Main Mall, Vancouver, British Columbia, V6T 1Z1, Canada*

E-mail: gcampillo@reed.edu

S1 Experimental information

Instruments and methods

Fluorescence spectra (excitation and emission) were obtained using a PTI QuantaMaster 400 fluorometer (Horiba) of suspended single crystals in heptane. The samples were stirred during measurements.

S2 Single-crystal X-ray diffraction data

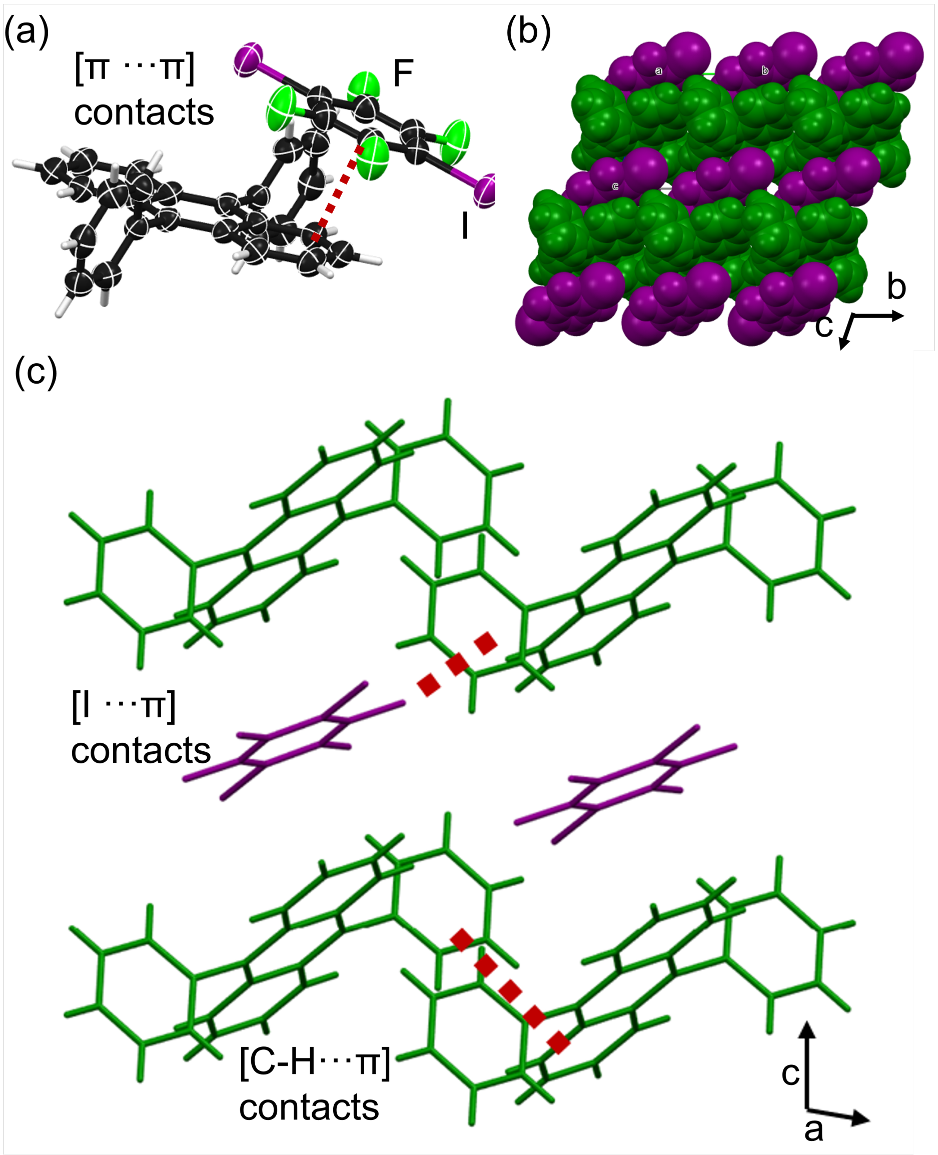

Figure S1: X-ray structure of **DPA·1,4-$C_6I_2F_4$**: (a) two-component assembly supported by [$\pi \cdots \pi$] contacts, (b) space-filling view of corrugated sheets along the *b*-axis, and (c) 1:1 mixed stacks supported by [C-I$\cdots\pi$] contacts and [C-H$\cdots\pi$] contacts in the *ac*-plane

Crystallographic tables

Table S1: Crystallographic parameters for **DPA·1,2-$C_6I_2F_4$**.

Parameter	Value
Compound name	**DPA·1,2-$C_6I_2F_4$**
Empirical formula	$C_{19}H_9F_4I_2$
Formula weight	567.06
Temperature / K	299(2)
Crystal system	triclinic
Space group	P-1
a/Å	8.3926(5)
b/Å	8.9190(5)
c/Å	12.6724(7)
$\alpha/^\circ$	89.703(4)
$\beta/^\circ$	71.340(5)
$\gamma/^\circ$	78.561(5)
Volume / Å^3	879.12(9)
Z	2
ρ_{calc}/g cm^{-3}	2.142
μ/mm^{-1}	3.615
$F(000)$	530.0
Crystal size	1 × 0.15 × 0.15 mm^3
Radiation	Mo Kα (λ = 0.71073 Å)
2Θ range / °	4.668–61.154
Index ranges	$-12 \leq h \leq 11,\ -12 \leq k \leq 12,\ -18 \leq l \leq 18$
Reflections collected	14773
Independent reflections	5064 [R_{int}=0.0836, R_{sigma}=0.0667]
Data/restraints/parameters	5064/0/227
Goodness-of-fit on F^2	1.034
Final R [I$\geq 2\sigma(I)$]	R_1=0.0551, wR_2=0.1365
Final R [all data]	R_1=0.0737, wR_2=0.1483
CCDC ID	2492528

Table S2: Crystallographic parameters for **DPA·1,3,5-$C_6I_3F_3$**.

Parameter	Value
Compound name	**DPA·1,3,5-$C_6I_3F_3$**
Empirical formula	$C_{25}H_9F_6I_6$
Formula weight	1184.72
Temperature / K	295.62(10)
Crystal system	triclinic
Space group	P-1
a/Å	9.1664(4)
b/Å	9.3672(4)
c/Å	17.0767(10)
$\alpha/^\circ$	87.224(4)
$\beta/^\circ$	88.576(4)
$\gamma/^\circ$	87.254(4)
Volume / Å^3	1462.49(12)
Z	2
ρ_{calc}/g cm^{-3}	2.690
μ/mm^{-1}	6.425
$F(000)$	1062.0
Crystal size	0.45 × 0.09 × 0.09 mm^3
Radiation	Mo Kα (λ = 0.71073 Å)
2Θ range / °	4.668–61.154
Index ranges	$-13 \leq h \leq 13,\ -13 \leq k \leq 12,\ -24 \leq l \leq 24$
Reflections collected	24843
Independent reflections	8486 [R_{int}=0.1249, R_{sigma}=0.1057]
Data/restraints/parameters	8486/0/335
Goodness-of-fit on F^2	0.943
Final R [I≥ $2\sigma(I)$]	R_1=0.0651, wR_2=0.1547
Final R [all data]	R_1=0.1108, wR_2=0.1876
CCDC ID	2492529

Table S3: Crystallographic parameters for **DPyA·1,2-$C_6I_2F_4$**.

Parameter	Value
Compound name	**DPyA·1,2-$C_6I_2F_4$**
Empirical formula	$C_{15}H_8F_2IN$
Formula weight	367.12
Temperature / K	278(10)
Crystal system	monoclinic
Space group	C2/c
a/Å	22.400(3)
b/Å	9.5167(7)
c/Å	15.586(2)
$\alpha/^\circ$	90
$\beta/^\circ$	126.48(2)
$\gamma/^\circ$	90
Volume / $Å^3$	2671.5(8)
Z	8
ρ_{calc}/g cm^{-3}	1.826
μ/mm^{-1}	2.405
$F(000)$	1408.0
Crystal size	0.28× 0.27 × 0.18 mm^3
Radiation	Mo Kα (λ = 0.71073 Å)
2Θ range / °	4.524–61.368
Index ranges	$-31 \leq h \leq 31,\ -12 \leq k \leq 13,\ -21 \leq l \leq 21$
Reflections collected	12670
Independent reflections	3904 [R_{int}=0.0687, R_{sigma}=0.0632]
Data/restraints/parameters	3904/0/173
Goodness-of-fit on F^2	0.981
Final R [I≥ $2\sigma(I)$]	R_1=0.0380, wR_2=0.0804
Final R [all data]	R_1=0.0617, wR_2=0.0902
CCDC ID	2492530

Table S4: Crystallographic parameters for **DPyA·1,4-$C_6I_2F_4$**.

Parameter	Value
Compound name	**DPyA·1,4-$C_6I_2F_4$**
Empirical formula	$C_{15}H_8F_2IN$
Formula weight	367.12
Temperature / K	296.15
Crystal system	monoclinic
Space group	C2/m
a/Å	16.1318(16)
b/Å	10.0496(12)
c/Å	8.0077(8)
α/°	90
β/°	97.346(9)
γ/°	90
Volume / Å^3	1287.5(2)
Z	4
ρ_{calc}/g cm^{-3}	1.894
μ/mm^{-1}	2.495
$F(000)$	704.0
Crystal size	1.21× 0.31 × 0.26 mm^3
Radiation	Mo Kα (λ = 0.71073 Å)
2Θ range / °	4.786–61.318
Index ranges	$-22 \leq h \leq 22,\ -13 \leq k \leq 14,\ -11 \leq l \leq 11$
Reflections collected	4815
Independent reflections	1975 [R_{int}=0.0614, R_{sigma}=0.0702]
Data/restraints/parameters	1975/0/144
Goodness-of-fit on F^2	1.070
Final R [I≥ $2\sigma(I)$]	R_1=0.0555, wR_2=0.1322
Final R [all data]	R_1=0.0820, wR_2=0.1456
CCDC ID	2492531

Table S5: Crystallographic parameters for **DPyA·1,3,5-$C_6I_3F_3$**.

Parameter	Value
Compound name	**DPyA·1,3,5-$C_6I_3F_3$**
Empirical formula	$C_{18}H_8F_3I_3N$
Formula weight	675.95
Temperature / K	296.4(2)
Crystal system	triclinic
Space group	P-1
a/Å	9.0031(6)
b/Å	9.5053(4)
c/Å	12.3397(7)
$\alpha/^\circ$	71.749(4)
$\beta/^\circ$	72.564(6)
$\gamma/^\circ$	82.252(4)
Volume / Å^3	955.82(10)
Z	2
$\rho_{\text{calc}}/\text{g cm}^{-3}$	2.349
μ/mm^{-1}	4.933
$F(000)$	618.0
Crystal size	$0.2\times$ 0.18 $\times$ 0.06 mm^3
Radiation	Mo Kα (λ = 0.71073 Å)
2Θ range / °	3.614–61.392
Index ranges	$-12 \leq h \leq 12,\ -13 \leq k \leq 13,\ -17 \leq l \leq 17$
Reflections collected	15092
Independent reflections	5520 [R_{int}=0.0348, R_{sigma}=0.0367]
Data/restraints/parameters	5520/0/226
Goodness-of-fit on F^2	1.050
Final R [I$\geq 2\sigma(I)$]	R_1=0.0327, wR_2=0.0716
Final R [all data]	R_1=0.0564, wR_2=0.0796
CCDC ID	2492532

Table S6: Selected intermolecular interaction energies for **DPA** and **DPyA** co-crystals obtained from CrystalExplorer calculations.

Crystal / Pair	Distance (Å)[a]	Description	E_{tot} (kJ mol^{-1})[b]	Symmetry code[c]
**DPA·1,4-$C_6I_2F_4$**				
DPA···1,4-$C_6I_2F_4$	5.16	$\pi\cdots\pi$	-43.2	1A:2A1x,y,1+z
DPA···DPA	7.88	C–H···π	-32.5	2A:2A-1+x,y,1+z
DPA···DPA	11.34	C–H···π	-15.6	2A:2A-1+x,-1y,z
DPA···1,4-$C_6I_2F_4$	9.02	C–I···π	-20.4	1A:2A2+x,y,1+z
**DPA·1,2-$C_6I_2F_4$**				
DPA···1,2-$C_6I_2F_4$	5.03	$\pi\cdots\pi$	-42.5	1A:2A
DPA···DPA	8.39	C–H···π	-25.3	2A:2A-1+x,y,z
DPA···DPA	10.97	$\pi\cdots\pi$	-18.8	2A:2A-1+x,1+y,z
DPA···1,2-$C_6I_2F_4$	9.32	C–I···π	-13.7	1A:2A-1+x,y,z
1,2-$C_6I_2F_4$···1,2-$C_6I_2F_4$	3.78	$\pi\cdots\pi$	-34.3	1A:1A1-x,1-y,2-z[d]
**DPA·1,3,5-$C_6I_3F_3$**				
DPA···1,3,5-$C_6I_3F_3$	4.49	$\pi\cdots\pi$	-51.5	1B:2A
DPA···1,3,5-$C_6I_3F_3$	9.20	C–I···π	-19.5	1B:2A1+x,y,z
DPA···DPA	9.37	C–H···π	-28.1	2A:2Ax,-1+y,z
1,3,5-$C_6I_3F_3$···1,3,5-$C_6I_3F_3$	9.36	I···I	-6.6	1B:1Bx,-1+y,z[d]
1,3,5-$C_6I_3F_3$···1,3,5-$C_6I_3F_3$	5.45	$\pi\cdots$F	-27.2	1A:1B[d]
**DPyA·1,2-$C_6I_2F_4$**				
DPyA···1,2-$C_6I_2F_4$	5.71	$\pi\cdots\pi$	-34.4	1A:2A1-x,-y,1-z
DPyA···1,2-$C_6I_2F_4$	11.83	C–I···N	-19.7	1A:2A
DPyA···DPyA	9.52	C–H···π	-19.6	2A:2Ax,-1+y,z
DPyA···DPyA	9.13	C–H···π	-20.2	2A:2Ax,-y,1/2+z
**DPyA·1,3,5-$C_6I_3F_3$**				
DPyA···1,3,5-$C_6I_3F_3$	12.02	C–I···N	-19.1	1A:2A
DPyA···1,3,5-$C_6I_3F_3$	9.92	$\pi\cdots\pi$	-47.1	1A:2A-1+x,y,z
DPyA···DPyA	9.00	C–H···π	-19.6	2A:2A-1+x,y,z
DPyA···DPyA	9.51	C–H···π	-24.0	2A:2Ax,-1+y,z
1,3,5-$C_6I_3F_3$···1,3,5-$C_6I_3F_3$	3.81	$\pi\cdots\pi$	-37.5	1A:1A-x,-y,2-z[d]
1,3,5-$C_6I_3F_3$···1,3,5-$C_6I_3F_3$	8.68	I···I	-9.6	1A:1A-1-x,-y,2-z[d]
1,3,5-$C_6I_3F_3$···1,3,5-$C_6I_3F_3$	9.51	I···I	-1.5	1A:1Ax,-1+y,z[d]

Notes: [a]Centroid–centroid distance between interacting molecules. [b]Total interaction energies computed using CrystalExplorer25 (CE-HF, Hartree–Fock method with the 3-21G basis set).[1,2] [c]Selecting **DPA** or **DPyA** as the origin unless otherwise noted. [d]Selecting halogenated coformer as the origin.

S3 Molecular modeling data

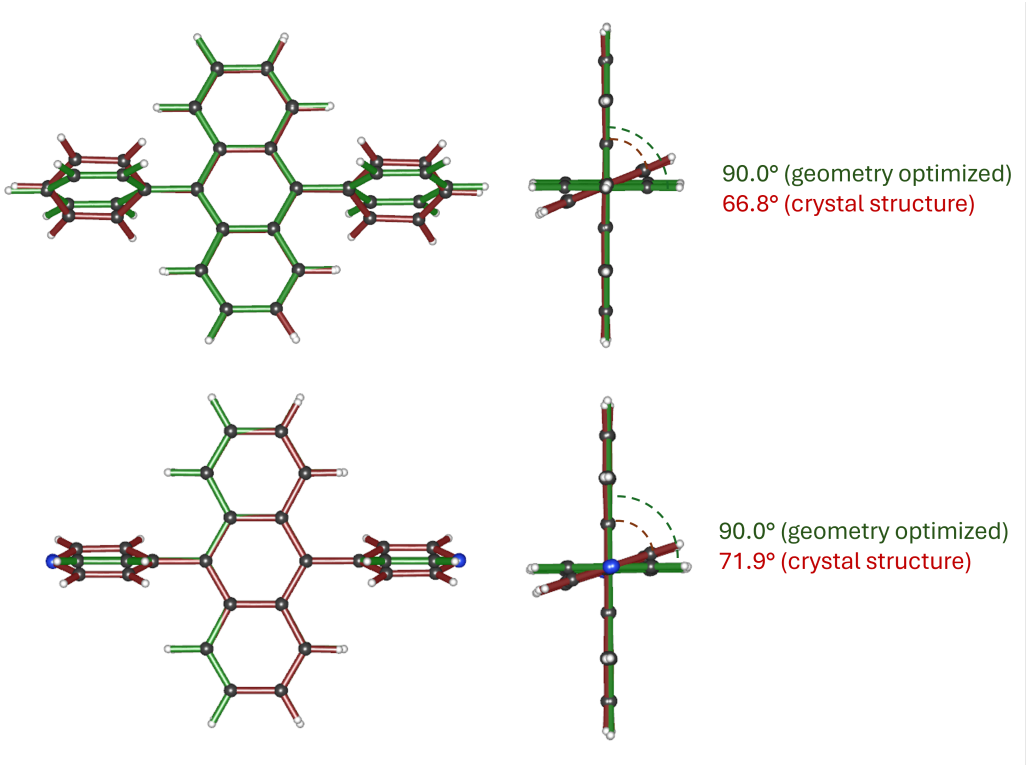

Figure S2: Molecular conformations of **DPA** (top) and **DPyA** (bottom). Green indicates conformation obtained by geometry optimization in Gaussian16 using Density Functional Theory (DFT) and the B3LYP/6-311G++(d,p) basis set.[3–5] Red indicates conformation extracted from crystallographic coordinates (refcodes: DPANTR01 and TUHWEZ for **DPA** and **DPyA**, respectively).[6,7]

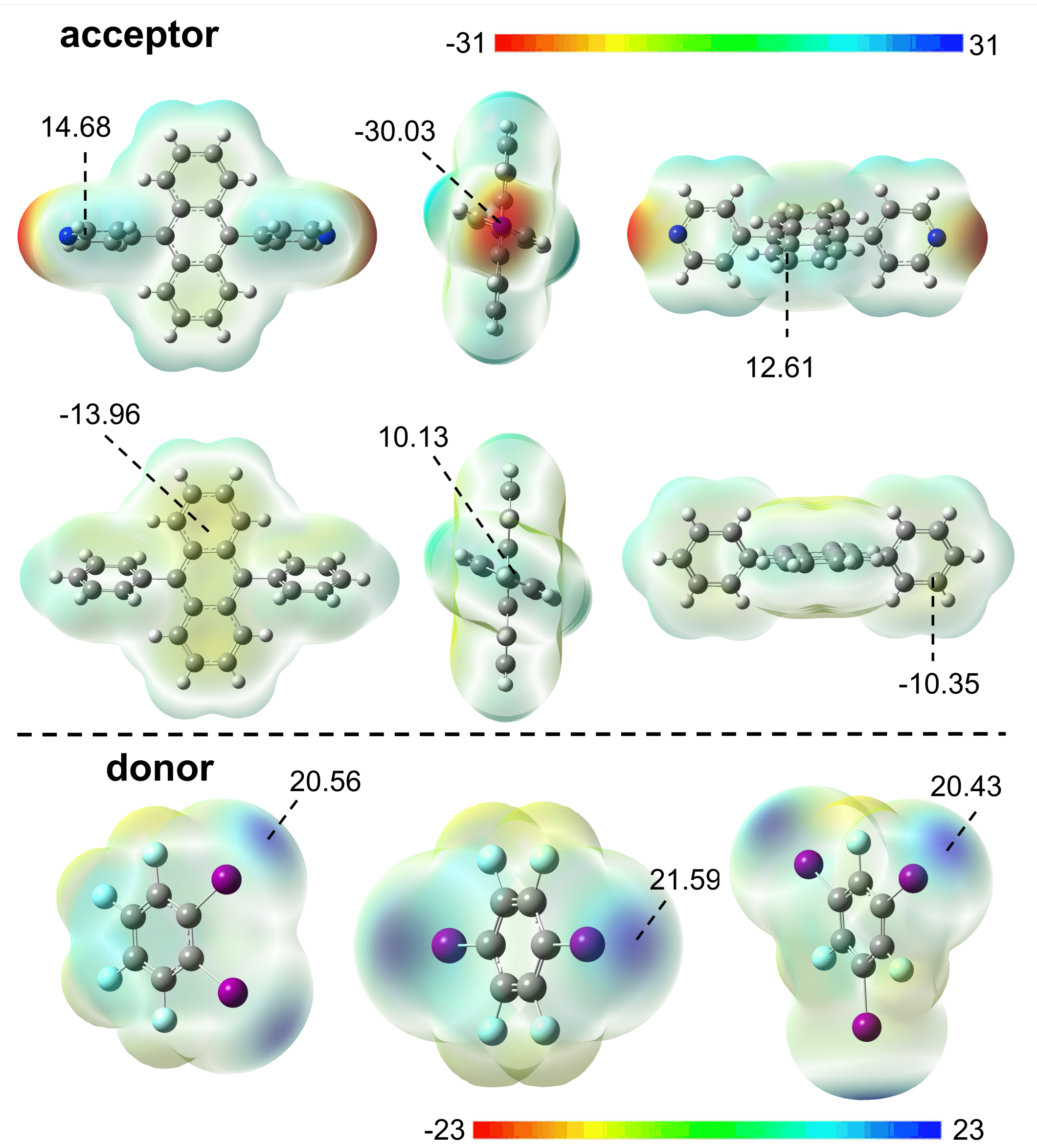

Figure S3: Electrostatic potential (ESP) maps from donor and acceptor molecules in this study. Maxima and minima values in marked regions are given in kcal/mol. ESP maps were calculated in Gaussian16 using DFT and the B3LYP/6-311G++(d,p) basis set for acceptor molecules and the 3-21G basis set for donor molecules.[3–5]

S4 Powder X-ray diffraction data

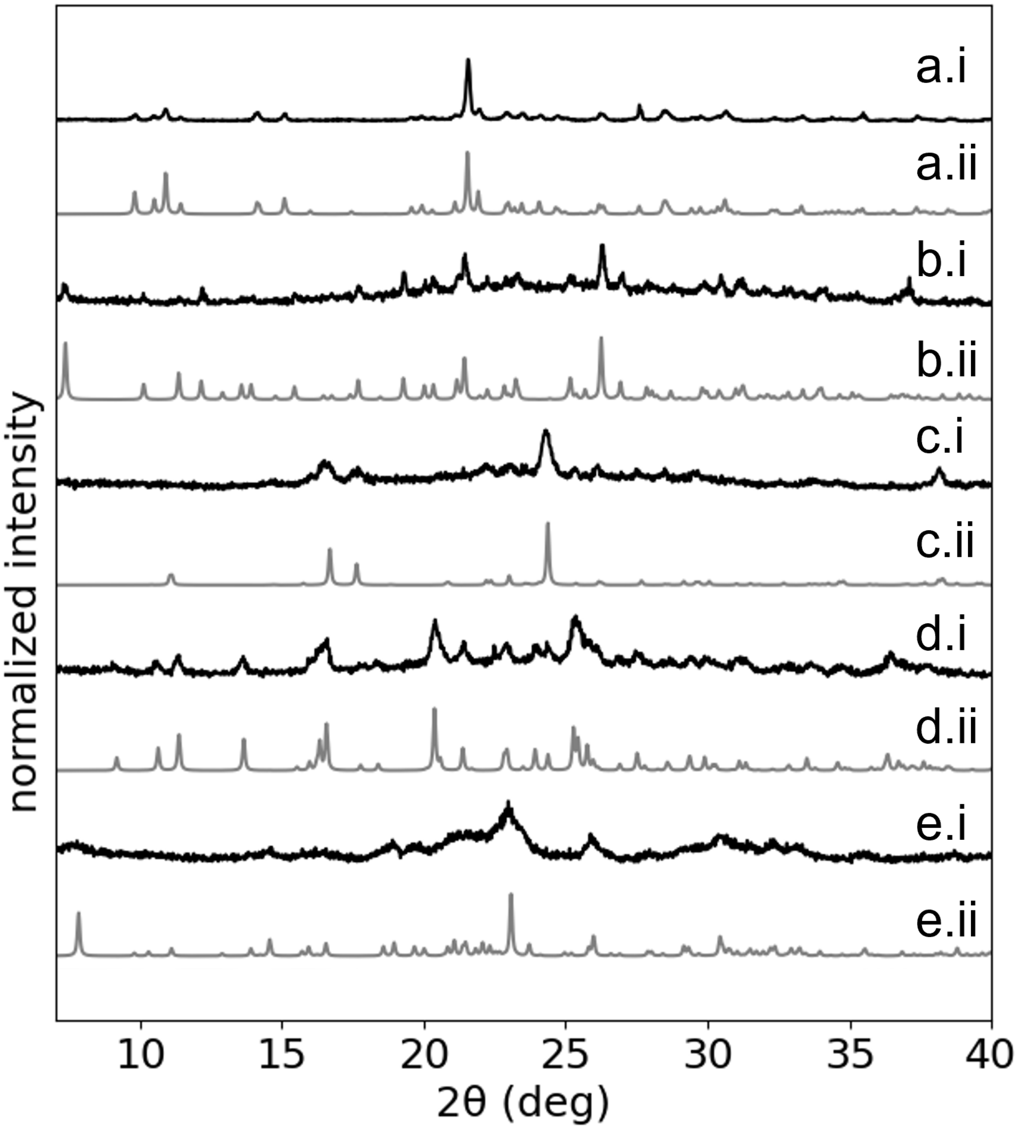

Figure S4: Experimental (i) and simulated (ii) PXRD patterns of co-crystals (a) **DPA·1,3,5-$C_6I_3F_3$**, (b) **DPyA·1,3,5-$C_6I_3F_3$**, (c) **DPA·1,4-$C_6I_2F_4$**, (d) **DPyA·1,4-$C_6I_2F_4$**, and (e) **DPA·1,2-$C_6I_2F_4$**.

S5 Photophysical data

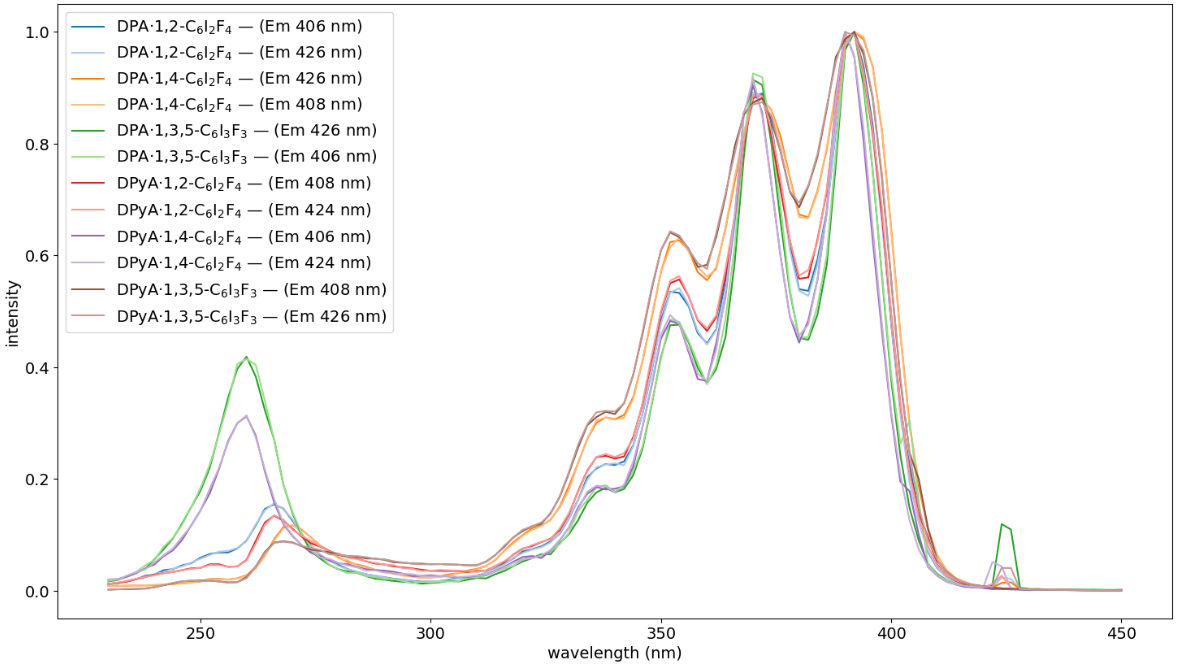

Figure S5: Excitation spectra of co-crystals of **DPA** and **DPyA** with halobenzenes.

References

(1) Spackman, P. R.; Turner, M. J.; McKinnon, J. J.; Wolff, S. K.; Grimwood, D. J.; Jayatilaka, D.; Spackman, M. A. CrystalExplorer: a program for Hirshfeld surface analysis, visualization and quantitative analysis of molecular crystals. *J. Appl. Crystallogr.* **2021**, *54*, 1006–1011.

(2) Mackenzie, C. F.; Spackman, P. R.; Jayatilaka, D.; Spackman, M. A. CrystalExplorer model energies and energy frameworks: extension to metal coordination compounds, organic salts, solvates and open-shell systems. *IUCrJ* **2017**, *4*, 575–587.

(3) Aakeröy, C. B.; Wijethunga, T. K.; Haj, M. A.; Desper, J.; Moore, C. The structural

landscape of heteroaryl-2-imidazoles: competing halogen-and hydrogen-bond interactions. *CrystEngComm* **2014**, *16*, 7218–7225.

(4) Zebbiche, Z.; Şekerci, G.; Houssem, B.; Küçükbay, F.; Tekin, S.; Küçükbay, H.; Boumoud, B. Synthesis and Biological Assessment of Cyanopyridine-Based 1, 3, 4-Oxadiazole Derivatives: Anticancer Potential, Antioxidant Activity, Molecular Docking, and DFT Calculations. *J. Biochem. Mol. Toxicol.* **2025**, *39*, e70346.

(5) Sambathkumar, K.; Nithiyanantham, S. Synthesis, characterization and theoretical properties of coumarin NLO single crystal by DFT method. *J. Mater. Sci.: Mater. Electron.* **2017**, *28*, 6529–6543.

(6) Langer, V.; Becker, H.-D. Crystal structure of 9, 10-diphenylanthracene,(C6H5)(C14H8)(C6H5). *Z. Kristallogr.* **1992**, *199*, 313–315.

(7) Cui, X.; Khlobystov, A. N.; Chen, X.; Marsh, D. H.; Blake, A. J.; Lewis, W.; Champness, N. R.; Roberts, C. J.; Schröder, M. Dynamic Equilibria in Solvent-Mediated Anion, Cation and Ligand Exchange in Transition-Metal Coordination Polymers: Solid-State Transfer or Recrystallisation? *Chem. Eur. J.* **2009**, *15*, 8861–8873.
